# Green manure increases peanut production by shaping the rhizosphere bacterial community and regulating soil metabolites under continuous peanut production systems

**DOI:** 10.1186/s12870-023-04079-0

**Published:** 2023-02-01

**Authors:** Yang Xu, Hong Ding, Guanchu Zhang, Zelun Li, Qing Guo, Hao Feng, Feifei Qin, Liangxiang Dai, Zhimeng Zhang

**Affiliations:** Shandong Peanut Research Institute, Shandong Academy of Agricultural Sciences, Qingdao, Shandong China

**Keywords:** Green manure, Bacterial community, Soil metabolites, Peanut yield, Continuous cropping

## Abstract

Green manure (GM) is a crop commonly grown during fallow periods, which has been applied in agriculture as a strategy to regulate nutrient cycling, improve organic matter, and enhance soil microbial biodiversity, but to date, few studies have examined the effects of GM treatments on rhizosphere soil bacterial community and soil metabolites from continuous cropping peanut field. Results: In this study, we found that the abundances of several functionally significant bacterial groups containing Actinobacteria, Acidobacteria, and genus *Sphingomonas*, which are associated with nitrogen cycling, were dramatically increased in GM-applied soils. Consistent with the bacterial community results, metabolomics analysis revealed a strong perturbation of nitrogen- or carbon-related metabolisms in GM-applied soils. The substantially up-regulated beneficial metabolites including sucrose, adenine, lysophosphatidylcholine (LPC), malic acid, and betaines in GM-applied soils may contribute to overcome continuous cropping obstacle. In contrast to peanut continuous cropping, planting winter wheat and oilseed rape in winter fallow period under continuous spring peanut production systems evidently improved the soil quality, concomitantly with raised peanut pod yield by 32.93% and 25.20%, in the 2020 season, respectively. Conclusions: GMs application is an effective strategy to overcome continuous cropping obstacle under continuous peanut production systems by improving nutrient cycling, soil metabolites, and rhizobacterial properties.

**Supplementary Information:**

The online version contains supplementary material available at 10.1186/s12870-023-04079-0.

## Background

Peanuts (*Arachis hypogaea* L.) as one of the most important oil seed crops are cultivated worldwide, which serves as a good source of protein, calories, vitamins, and minerals for human beings [1–6]. China is the largest peanut producer globally, whereas peanut production currently faces challenges due to long-term continuous cropping [7]. Continuous cropping usually inhibits plant growth, reduces yield, and causes continuous cropping obstacles, which results in severe economic losses and hinders sustainable crop industry development. Continuous cropping obstacles are generally due to the deterioration of soil physiochemical properties, disturbance of the native soil microbiota, or development of soil-borne pathogens [7–10]. Therefore, overcoming continuous cropping obstacles, improving the soil quality, and modifying the soil microbiota in peanut continuous cropping systems are critical to sustainable peanut production.

Soil production ability depends on nutrient cycling, soil structure, and soil microbial biodiversity. While cultivation can reduce the organic matter content and change soil microbial community structure, which is aggravated by continuous cropping [11, 12]. Green manure (GM) is a crop commonly grown during fallow periods, which has been applied in agriculture as a strategy to regulate nutrient cycling, improve soil organic matter, and enhance soil microbial biodiversity [13]. The mexican sunflower (*Tithonia diversifolia Asteraceae*) was used as GM due to its relatively high nutrient concentrations (nitrogen, phosphorus, and potassium) and rapid decomposition to increase tiger nut (*Cyperus esculentus* L.) yield [14]. Alfalfa (*Medicago sativa* L.) as GM had a great ability to increase rice grain yield by increasing soil labile phosphorus fractions, phosphorus uptake, and soil enzyme activities [15]. Using Chinese milk vetch (*Astragalus sinicus* L.) as GM significantly increased the rice yield by improving NH_4_^+^ content in soil [16]. Wheat (*Triticum aestivum* L.) as GM significantly changed the abundance of Xanthobacteraceae family of Proteobacteria, which increased soil nitrogen concentration, improving the peanut yields under continuous spring peanut production systems [7]. Moreover, the incorporation of plant biomass into soil as GMs can reduce the abundance of the soilborne plant pathogen *Verticillium dahliae* and increase the abundance of bacterial functional traits related to iron and polysaccharide acquisition in rhizosphere soil [17]. Thus, GMs application as a useful management practice can improve soil nutrient-supplying capacity and soil microbial community structure and to increase the crop yield; however, few studies have estimated how GMs application affected changes in soil metabolites.

The soil ecosystem functions consist of physical, chemical, and biological characteristics linked to soil microbes, metabolites, and extracellular enzymes activities [18]. Soil metabolites determination is widely used to evaluate changes in soil quality and microbial community, because variations experienced at the microbial and enzyme levels will be manifested at the metabolite level [19, 20]. Comprehensive analysis of the soil rhizobacterial community assembly and concurrent assessment of changes in the soil metabolites is a good strategy to evaluate soil productivity [21, 22]. However, the integrated analysis of rhizobacterial structure and associated metabolites in soils planting GMs in winter fallow period under continuous spring peanut production systems is lacking. Here, we evaluated the effects of different GMs on soil rhizobacterial community diversity, metabolites changes, and peanut pod yields to identify an optimal GM crop for addressing the challenges arising from the continuous cropping obstacle under continuous peanut production systems. We propose that GM application with winter wheat may be better than oilseed rape to overcome continuous cropping obstacle under continuous spring peanut production systems.

## Results

### GM treatments increase peanut yield

Notably, the application of GMs in winter fallow improved peanut growth and pod yields in the next growing season (Table 1). Significant differences were observed in 100 pods weight, 100 seeds weight, pods per plant, and pod yield among the various treatments (Table 1). As expected, the treatments of planting winter wheat as GMs in winter fallow (WW) and planting oilseed rape as GMs (OR) increased pod yields notably by increasing the pods per plant, 100 pods weight, and 100 seeds weight as compared with those in the peanut continuous cropping (CC). The highest yield among all the treatments was observed in the WW-applied plots, followed by OR-applied plots. Compared with those in the CC treatment, peanut pod yields were 32.93% and 25.20% higher in the WW and OR treatments, respectively, in the 2020 season. These data strongly suggest that GM with winter wheat is better than with oilseed rape in winter fallow period under continuous peanut production systems.

**Table 1:** Effects of GMs application on morphology and pod yield of peanuts

Year	Treatment	Main stem height	Lateral branch length	Pods per plant	100 pods weight (g)	100 seeds weight (g)	Kernel rate to pod (%)	Pod yield (kg hm^−2^)
2019	CC	37.33 ± 2.32c	39.33 ± 3.06c	20 ± 4c	117.5 ± 2.51b	76.2 ± 1.59b	61.2 ± 1.06b	3086.15 ± 111.76c
	WW	55.33 ± 3.21a	61.03 ± 0.95a	35.09 ± 0.82a	156.6 ± 2.97a	88.46 ± 2.17a	67.23 ± 0.71ab	4072.3 ± 94.83a
	OR	53.6 ± 1.51ab	54 ± 1.73b	28.5 ± 1.5b	154.1 ± 5.18a	87.5 ± 2.5a	68.13 ± 1.8a	3751 ± 39.15b
2020	CC	37.5 ± 1c	40.67 ± 1.29bc	13.5 ± 1.5c	118.85 ± 1.43b	75.33 ± 1.69b	60.36 ± 1.87b	2850.55 ± 36.27c
	WW	53.33 ± 0.79a	56.17 ± 0.98a	26.5 ± 0.66a	156.62 ± 2.57a	86.35 ± 1.6a	65.38 ± 1.6a	3788.65 ± 118.43a
	OR	41.98 ± 1.81b	43.33 ± 2b	20.33 ± 4.04b	149.24 ± 2.42a	85.8 ± 3.16a	62.87 ± 1.74ab	3568.15 ± 186.03b
*P*								
Year (Y)		*	*	*	ns	ns	ns	*
Treatment (T)		*	*	*	*	*	*	*
Y × T		*	*	ns	ns	ns	ns	*

^*^ Significant at *P* < 0.05. ns: nonsignificant

### Alpha and beta diversity analysis of the rhizosphere and bulk soils

**Fig. 1:**
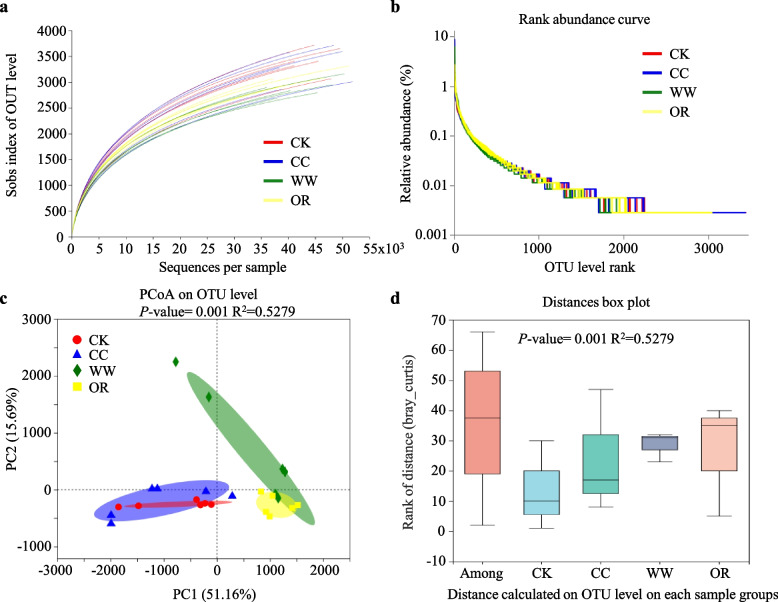
Alpha and beta diversity analysis. **a** Rarefaction curve analysis showing the 16S rRNA gene sequencing depth and the bacterial community diversity of the rhizosphere and bulk soils. **b** Rank abundance curves showing the relative species abundance and evenness of the rhizosphere and bulk soils. **c** Principal co-ordinates analysis (PCoA) analysis. The same color points belong to the same soil group, and the same soil group points are marked by ellipses. **d** Analysis of similarities (ANOSIM) analysis revealed the variation in the bacterial composition (Bray–Jaccard distance) of the rhizosphere and bulk soils

We performed alpha diversity analysis to evaluate bacterial community richness and diversity. Rarefaction curve analysis exhibited a high gene sequencing depth and a great possibility of observing community diversity since the rarefaction curves of soil samples did not approach the asymptote (Fig. 1a). Rank abundance curves showed long and flat polyline on the horizontal axis, indicating a high evenness and diversity of the bacterial community composition (Fig. 1b). We also performed beta diversity analysis to observe the similarities and dissimilarities among the soil samples. A significant difference in variance could be observed between soil groups in principal co-ordinates analysis (PCoA) analysis: GM-applied soil samples WW and OR showed an obvious separation of the bacterial community composition with CK and CC (Fig. 1c). This result was confirmed by analysis of similarities (ANOSIM) analysis (*P* < 0.001; Fig. 1d). The rhizosphere bacterial structures of GM-applied soil samples are separate from those of CK and CC, suggesting that the application of GMs affects the peanut rhizobacterial assembly.

### GMs application reshapes soil rhizosphere bacterial community

**Fig. 2:**
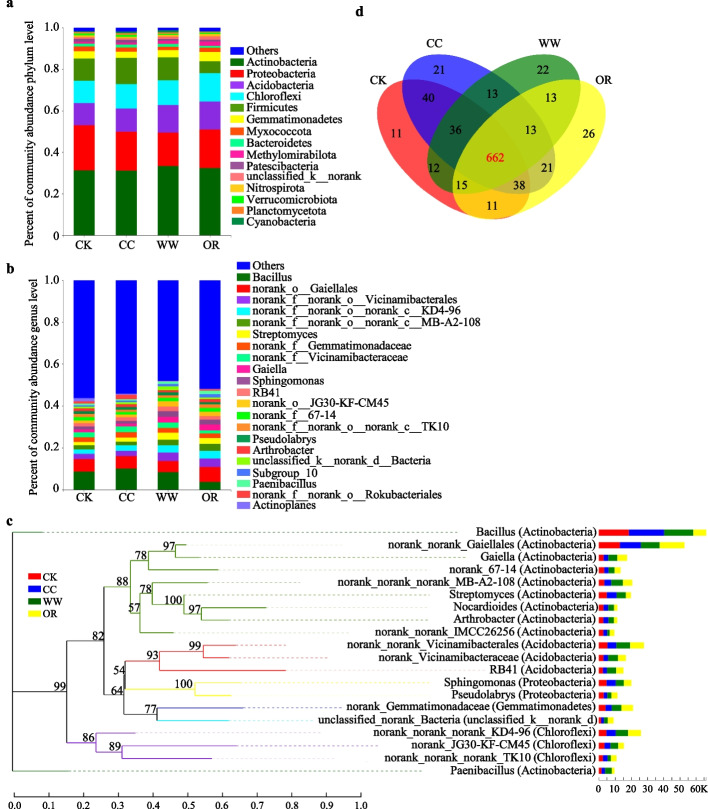
Bacterial community structure in the rhizosphere and bulk soils. **a** Percent of taxa at the phylum level in the rhizosphere and bulk soils. The relative abundance of each taxon was calculated by averaging the abundances of three duplicates in each soil group. **b** Percent of taxa at the genus level in the rhizosphere and bulk soils. **c** Phylogenetic tree showing the phylogenetic relationship of the bacterial community at the genus level. The box size on the right corresponds to the average relative abundance of the phylum in the corresponding soil. The phylum that the classification genus belonged to is marked in the braces behind the corresponding genus. **d** Venn diagrams representing the total numbers of OTUs in four soil groups and are shared with each other

To explore how GM regulates the bacterial community diversity, we estimated the specific composition of each soil sample at five levels of classification (phylum, class, order, family, and genus) (Fig. 2, Fig. S1). Although the abundance of phyla varied in the rhizosphere and bulk soil samples, Actinobacteria, Proteobacteria, Acidobacteria, Chloroflexi, and Firmicutes were the common dominant bacteria in all the soil groups, accounting for over 80% of the bacterial taxa (Fig. 2a). Among them, Actinobacteria increased by 7.25% ~ 8.13% and 3.86% ~ 4.70% in the WW and OR compared to these of the CK and CC, and Acidobacteria increased by 15.78% ~ 20.90% and 18.26% ~ 23.29% in the WW and OR, respectively. However, the quantities of Firmicutes decreased in these soils, especially in OR (Fig. 2a). Moreover, Methylomirabilota also exhibited higher abundance in WW and OR in comparison with CC. The relative abundance of all bacterial genera was less than 10% at the genus level, indicating a high diversity of bacterial community at this level (Fig. 2b). The abundance of *norank_f__norank_o__Vicinamibacterales*, *norank_f__norank_o__norank_c__KD4-96*, *Streptomyces*, and *Sphingomonas* notably increased in GM-applied soils, whereas *Bacillus* showed the contrary trend (Fig. 2b). Among them, *Streptomyces* and *Sphingomonas* exhibited higher abundance in WW than those in OR. In addition, the unclassified or unnamed sequences were abundant in these soil samples, demonstrating that the soil remains a challenging reservoir of bacterial diversity (Fig. 2b).

Phylogenetic tree analysis showed that the top 20 most abundant genera belonged to the phyla of Actinobacteria (10/20), Acidobacteria (3/20), Chloroflexi (3/20), Proteobacteria (2/20), Gemmatimonadetes (1/20), and unclassified_k__norank_d__Bacteria (1/20) (the proportion of genera in the specific phylum was shown in brackets) (Fig. 2c). Moreover, Venn diagrams revealed that the distribution of the genus of the bacterial communities in the four soil groups was diverse (Fig. 2d). The genus numbers of CK, CC, WW, and OR were 825, 844, 786, and 799, and there were 662 common genera across the four soil groups, accounting for approximately 69.39% of the total numbers of genera. Furthermore, the unique numbers of genera in CK, CC, WW, and OR were 11, 21, 22, and 26, respectively (Fig. 2d). Thirteen specific genera were detected in CC, WW, and OR, but none in CK (Fig. 2d). Thus, the application of winter wheat and oilseed rape reshaped the rhizosphere bacterial community structure, and the bacterial abundance was unique to some individuals.

### The application of GMs alters soil metabolic profiles

**Fig. 3:**
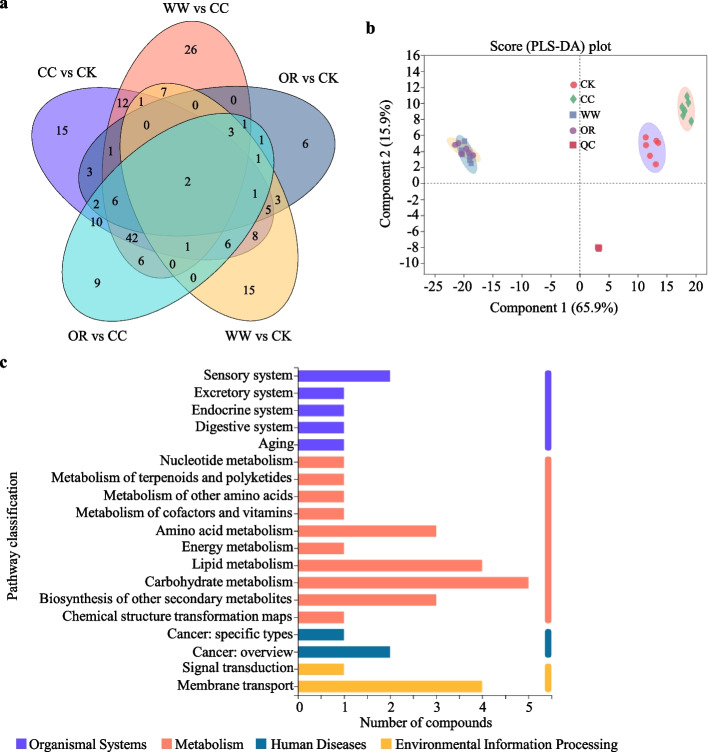
The variation of soil metabolic profiles. **a** Venn analysis of the soil metabolites that significantly differed in relative abundance in CC vs CK, OR vs CK, WW vs CK, OR vs CC, and WW vs CC. Each circle in the graph represents one group. **b** Partial least-squares-discriminant analysis (PLS-DA) of the soil metabolic profiles. **c** KEGG pathway classification of the differentially expressed metabolites

Through liquid chromatography-mass spectrometry-based nontargeted metabolomics analysis, we identified 260 and 198 metabolites in soils in positive and negative ion mode, respectively. Among them, a total of 68 and 40 metabolites were identified and named based on the Kyoto Encyclopedia of Genes and Genomes (KEGG), respectively (Fig. 3, Table S1 and S2). The Venn diagram demonstrated the unique metabolites with substantial differences between CC vs CK, OR vs CK, WW vs CK, OR vs CC, and WW vs CC were 15, 6, 15, 9, and 26 (Fig. 3a). Among them, 2 metabolites coexisted in all the soil groups (Fig. 3a). The partial least-squares discrimination analysis (PLS-DA) demonstrated a clear separation between GM-applied soils and CK. CC also showed a substantial difference in variance with CK, indicating that both GMs application and continuous cropping altered the soil metabolites and different soil types exhibited different responses (Fig. 3b). The quality control (QC) samples in the positive and negative ion modes were closely clustered, indicating that the experiments were repeatable and that the test data were stable and reliable (Fig. 3b).

Variable importance in projection (VIP) values in the PLS-DA model were calculated to detect the changes in soil metabolites in detail. Metabolites with VIP values > 1.0 and *P*-values < 0.05 (*t*-test) were considered significantly affected by GM treatments. The results revealed a significant difference in the metabolites produced by the GM-applied soils and CC. Among them, raffinose, melibiose, stachyose, 3-dehydrosphinganine, betaine, trimethylselenonium, sucrose, 2-methoxyestradiol-17beta 3-sulfate, adenine, lysophosphatidylcholine (LPC), and malic acid were dramatically elevated in WW and OR compared to those of CC, whereas 3-aminopropanal and hypoxanthine were only increased in OR. Some metabolites exhibited the opposite trends, 7,8-dihydroneopterin, daidzein, and resveratrol decreased in WW, while 7,8-dihydroneopterin and sucralose declined in OR as comparison with those of CC (Table 2). This indicates that the application of GMs altered soil metabolites, and different GMs produced diverse metabolites.

**Table 2:** Differentially expressed metabolites identified in KEGG pathway of WW vs CC and OR vs CC

Differentially expressed metabolites		Up or down	KEGG pathway Description
WW vs CC	OR vs CC		
Raffinose	Raffinose	Up	Metabolic pathways; ABC transporters; Galactose metabolism
Melibiose	Melibiose	Up	Metabolic pathways; ABC transporters; Galactose metabolism
Stachyose	Stachyose	Up	Metabolic pathways; Galactose metabolism
3-Dehydrosphinganine	3-Dehydrosphinganine	Up	Sphingolipid metabolism; Metabolic pathways
Betaine	Betaine	Up	Metabolic pathways; ABC transporters; Glycine, serine and threonine metabolism
Trimethylselenonium	Trimethylselenonium	Up	Selenocompound metabolism
Sucrose	Sucrose	Up	Metabolic pathways; Biosynthesis of secondary metabolites; Carbohydrate digestion and absorption; ABC transporters; Galactose metabolism; Starch and sucrose metabolism; Phosphotransferase system (PTS); Taste transduction
2-Methoxyestradiol-17beta 3-sulfate	2-Methoxyestradiol-17beta 3-sulfate	Up	Steroid hormone biosynthesis
Adenine	Adenine	Up	Metabolic pathways; Purine metabolism; Zeatin biosynthesis
lysophosphatidylcholine (LPC)	lysophosphatidylcholine (LPC)	Up	Glycerophospholipid metabolism
Malic acid	Malic acid	Up	Biosynthesis of secondary metabolites; Renal cell carcinoma; Carbon metabolism; Pyruvate metabolism; Citrate cycle (TCA cycle); Glyoxylate and dicarboxylate metabolism; Proximal tubule bicarbonate reclamation; Carbon fixation in photosynthetic organisms; Glucagon signaling pathway; Biosynthesis of alkaloids derived from terpenoid and polyketide; Biosynthesis of alkaloids derived from histidine and purine; Taste transduction; Biosynthesis of alkaloids derived from shikimate pathway; Biosynthesis of terpenoids and steroids; Biosynthesis of phenylpropanoids; Biosynthesis of plant secondary metabolites; Metabolic pathways; Microbial metabolism in diverse environments; Methane metabolism; Biosynthesis of alkaloids derived from ornithine, lysine and nicotinic acid; Two-component system; Biosynthesis of plant hormones
/	3-Aminopropanal	Up	Metabolic pathways; beta-Alanine metabolism
/	Hypoxanthine	Up	Metabolic pathways; Purine metabolism
/	7,8-Dihydroneopterin	Down	Metabolic pathways; Folate biosynthesis
/	Sucralose	Down	Carbohydrate digestion and absorption
7,8-Dihydroneopterin	/	Down	Metabolic pathways; Folate biosynthesis
Daidzein	/	Down	Isoflavonoid biosynthesis; Biosynthesis of secondary metabolites; Biosynthesis of phenylpropanoids
Resveratrol	/	Down	Biosynthesis of secondary metabolites; Biosynthesis of phenylpropanoids; Stilbenoid, diarylheptanoid and gingerol biosynthesis

As comparison with CC, carbohydrate metabolism, lipid metabolism, biosynthesis of secondary metabolites, amino acid metabolism, and membrane transport were the top 5 differentially expressed metabolites enriched KEGG pathways correlated with GM treatments (Fig. 3c, Table 3, Fig. S2). Most of the enriched KEGG pathways were identical in the two GM-applied soils, only a small number of pathways were diverse, such as beta-alanine metabolism, alpha-linolenic acid metabolism, biotin metabolism, and tropane, piperidine and pyridine alkaloid biosynthesis were only detected in OR. In contrast, folate biosynthesis, flavonoid biosynthesis, stilbenoid diarylheptanoid, and gingerol biosynthesis were just found in WW compared with those of CC (Table 3). These data further suggest that the application of GMs altered soil metabolic profiles.

**Table 3:** KEGG pathway analysis of WW vs CC and OR vs CC

KEGG pathway	
WW vs CC	OR vs CC
Steroid hormone biosynthesis	Steroid hormone biosynthesis
Purine metabolism	Purine metabolism
Selenocompound metabolism	Selenocompound metabolism
Pyruvate metabolism	Pyruvate metabolism
Biosynthesis of secondary metabolites	Biosynthesis of secondary metabolites
Zeatin biosynthesis	Zeatin biosynthesis
Isoflavonoid biosynthesis	Isoflavonoid biosynthesis
Biosynthesis of phenylpropanoids	Biosynthesis of phenylpropanoids
Biosynthesis of terpenoids and steroids	Biosynthesis of terpenoids and steroids
Biosynthesis of alkaloids derived from shikimate pathway	Biosynthesis of alkaloids derived from shikimate pathway
Biosynthesis of alkaloids derived from ornithine, lysine and nicotinic acid	Biosynthesis of alkaloids derived from ornithine, lysine and nicotinic acid
Biosynthesis of alkaloids derived from histidine and purine	Biosynthesis of alkaloids derived from histidine and purine
Biosynthesis of alkaloids derived from terpenoid and polyketide	Biosynthesis of alkaloids derived from terpenoid and polyketide
Biosynthesis of plant hormones	Biosynthesis of plant hormones
Methane metabolism	Methane metabolism
Glycine, serine and threonine metabolism	Glycine, serine and threonine metabolism
Citrate cycle (TCA cycle)	Citrate cycle (TCA cycle)
Glyoxylate and dicarboxylate metabolism	Glyoxylate and dicarboxylate metabolism
Carbon metabolism	Carbon metabolism
Galactose metabolism	Galactose metabolism
Starch and sucrose metabolism	Starch and sucrose metabolism
Glycerophospholipid metabolism	Glycerophospholipid metabolism
Sphingolipid metabolism	Sphingolipid metabolism
ABC transporters	ABC transporters
/	beta-Alanine metabolism
/	alpha-Linolenic acid metabolism
/	Biotin metabolism
/	Tropane, piperidine and pyridine alkaloid biosynthesis
Folate biosynthesis	/
Flavonoid biosynthesis	/
Stilbenoid, diarylheptanoid and gingerol biosynthesis	/

### Correlation between bacterial communities and soil metabolites

**Fig. 4:**
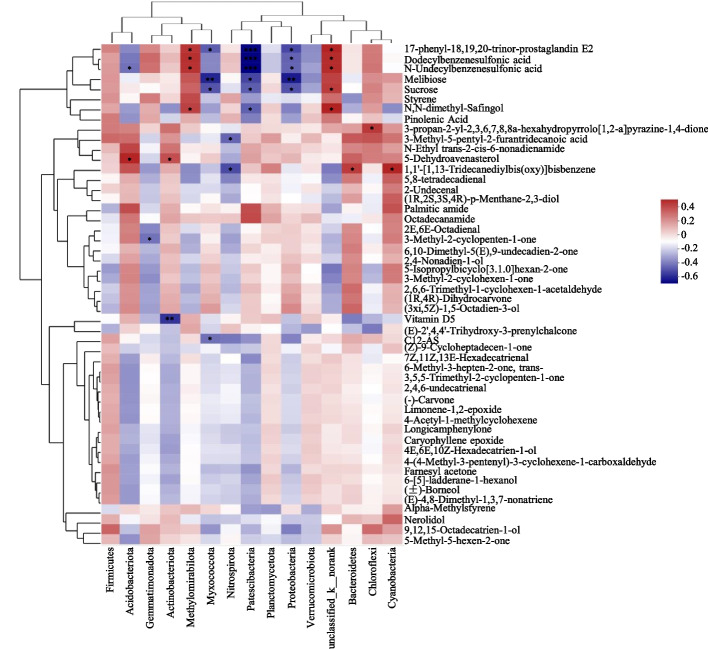
Correlation analysis between bacteria and soil metabolites. Red boxes represent positive correlations, while blue boxes represent negative correlations (Pearson’s correlation, *n* = 6, *P* < 0.05). Black asterisks indicate statistical significance: *, *P* < 0.05; **, *P* < 0.01; ***, *P* < 0.001

Correlations between rhizosphere soil bacteria and metabolites with significant differences among diverse treatments between 50 metabolites and 15 phyla were obtained via Pearson’s correlation analysis (*P* < 0.05; Fig. 4). Proteobacteria and Patescibacteria were negatively correlated with 17-phenyl-18,19,20-trinor-prostaglandin E2, dodecylbenzenesulfonic acid, N-undecylbenzenesulfonic acid, melibiose, ducrose, and N,N-dimethyl-Safingol, while p__unclassified_k__norank showed the opposite trend (Fig. 4). Furthermore, we determined that the relative abundances of Actinobacteria and Acidobacteria had a significantly positively correlation with 5-dehydroavenasterol that belongs to the biosynthesis of secondary metabolites and lipid metabolism through KEGG analysis (Fig. 4, Table 1). Nitrospirota showed a strongly negative correlation to 1,1'-[1,13-Tridecanediylbis(oxy)]bisbenzene, while Bacteroidota and Cyanobacteria had a significantly positive correlation with this metabolite (*P* < 0.05; Fig. 4). Moreover, 17-phenyl-18,19,20-trinor-prostaglandin E2, dodecylbenzenesulfonic acid, N-undecylbenzenesulfonic acid, and N,N-dimethyl-Safingol were also positively correlated with methylomirabilota. In addition, myxococcota showed negative correlation to the 17-phenyl-18,19,20-trinor-prostaglandin E2, melibiose, sucrose, and C12-AS (Fig. 4). Correlation heatmap analysis further exhibited strong interrelationships of bacterial taxa with soil metabolites in rhizosphere soils. As the previous study, the positive co-occurrence indicated that metabolites might secrete by microbes, and negative co-occurrence might be due to specific microbial consumption or degradation [22]. These data support that soil bacterial communities may be the main driver for the alterations in soil metabolic profiles, and the changing some certain metabolic pathways may be a primary strategy for soil bacterial communities to adapt to external environment.

### Effects of GM treatments on soil enzyme activities and chemical properties

**Fig. 5:**
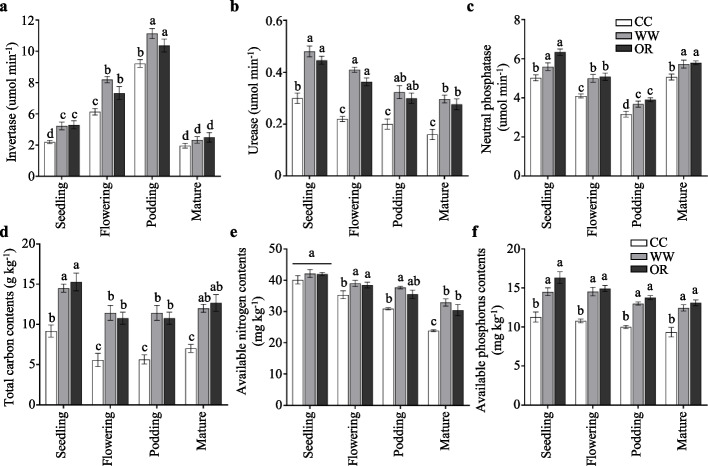
Soil enzyme activities and properties. Soil **a** invertase, **b** urease, **c** neutral phosphatase activities, **d** total carbon contents, **e** available nitrogen contents, and **f** available phosphorus contents of the rhizosphere and bulk soils. Error bars indicate the SEM (*n* = 3). One-way ANOVA Duncan’s test. Different lowercase letters represent different significance on the column

To determine whether extracellular enzyme activities were varied with the changes in the functional bacterial phyla in GM-applied rhizosphere soils, soil invertase, urease, and neutral phosphatase of different growth stages were measured (Figs. 5a-c). We found that invertase activity increased at first and then decrease in all the soil groups, and podding stage exhibited the highest (Fig. 5a), whereas the neutral phosphatase just showed the contrary trends (Fig. 5c). Soil urease activity decreased with the growth periods went on (Fig. 5b). All the soil enzymes were dramatically elevated in the GM-applied soils at various growth stages in compared with those in peanut continuous cropping soils. The average urease activity of WW and OR increased by 60.0% and 48.9%, and phosphatase activity raised by 11.3% and 25.8% compared with those of CC at the seedling stage, respectively (Figs. 5b, c). Soil urease is involved in nitrogen cycle and soil phosphatase can hydrolyze soil organic phosphate into inorganic phosphate for plants [23, 24]. Thus, the higher soil urease and phosphatase activities in GM-applied soils may improve soil production ability.

Then the soil chemical properties were examined. The soil total carbon contents and available nitrogen contents were evidently declining with the growth periods went on, whereas the available phosphorus contents showed no significant difference among different growth stages (Fig. 5d-f). The highest available nitrogen contents were observed in WW, and followed by OR at the flowering, podding, and mature stages (Fig. 5e). In contrast to peanut continuous cropping, available phosphorus contents occurred in relatively higher level in GM-applied soils at all four-growth stages, rising about 28.7% ~ 35.0% in WW and 37.7% ~ 45.0% in OR (Fig. 5f). These results suggest that apart from GM as an alternative to fertilizer, the (i) higher soil urease and phosphatase activities in GM-applied soils may be also conducive to improving soil nitrogen and phosphorus supply capacity, and (ii) GM treatments may be beneficial to overcome continuous cropping obstacle in peanut growth and production.

## Discussion

Soil fertility has deteriorated over the years by continuous cropping and GMs represent a promising approach to maintaining the sustainable nutrients for crop growth [15]. Indeed, GM treatments increased the peanut pod yield (Table 1). Comparably, winter wheat was superior to oilseed rape in overcoming continuous cropping obstacle and improving peanut yields. A previous study indicated that gramineous crops can offer several rotational benefits in single-season peanut cropping systems [25, 26]. Gramineous crops, especially wheat, remarkably improved available iron in rhizosphere soil, which further improves iron absorption for peanut [26]. We assumed that wheat-peanut (dicotyledon and monocotyledon, respectively) may have higher complementary than oilseed-peanut (both monocotyledons). Furthermore, our work suggests that the effects of winter wheat are better than oilseed rape in improving soil productivity and rhizosphere bacterial community. For example, winter wheat supplied more nitrogen source than oilseed rape for peanut (Fig. 5e); the elevation of carbon and nitrogen cycle-related bacteria (Actinobacteria and *Sphingomonas*) was more substantial in wheat-applied soils (Figs. 2a, b). Thus, we make an assumption that winter wheat may be better than oilseed rape as GMs for overcoming continuous cropping obstacle and improving peanut production by regulating soil nutrients, bacterial community composition, soil metabolites or some unknown manner.

GMs are beneficial for subsequent crops because of the nutrients that are released when their residues return to the soils [27]. Consistent with this, the soil total carbon contents, available nitrogen contents, and available phosphorus contents were dramatically higher in GM-applied soils (Figs. 5d-f). In addition, the higher soil urease and phosphatase activity in GM-applied soils (Figs. 5b, c) may contribute to nutrient cycling [23, 28]. Since soil tillage indeed increased soil urease and alkaline phosphatase activities, which, in turn, improved soil nutrient cycling [29]. Thus, apart from the GMs as residues returned to soils, the improved soil nutrients potentially also because the increased soil urease and phosphatase activities, further leading to the dramatically higher peanut pod yields in GM-applied soils.

Rhizosphere microbial communities benefit plants by increasing nutrient availability, producing plant growth hormones, or defending against pathogens [30–32]. In this study, there are many specific genera in CK, CC, WW, and OR, the unique to some individuals may be a result of specific root exudates of diverse treatments with or without GMs (Fig. 2d). The addition of GMs significantly enriched the population of bacteria potentially active in the soil nutrient cycle, containing Actinobacteria and Acidobacteria, and bacterial genus *Sphingomonas* (Figs. 2a, b). Actinobacteria are mostly aerobic saprophytes, which are widely distributed in various soils and have symbiotic nitrogen fixation and phosphorus solubilization [33]. The phylum Acidobacteria, one of the most widespread bacteria in the soils, exhibit an associative relationship to carbon and nitrogen cycle [34]. *Sphingomonas* are reported to play an important role in nitrogen fixation and denitrification in the nitrogen and carbon cycle [35, 36]. The relative higher abundance of the Actinobacteria and Acidobacteria, and *Sphingomonas* in rhizosphere soils of GMs application could explain the relatively higher available nitrogen and phosphorus concentration in these soils in the present study. Thus, GMs application alters rhizosphere bacterial communities and benefits plants by increasing nutrient availability. Our next step is to perform isolation and verification.

Several metabolites, including raffinose, melibiose, stachyose, 3-dehydrosphinganine, betaine, trimethylselenonium, sucrose, 2-methoxyestradiol-17beta 3-sulfate, adenine, LPC, and malic acid were significantly (*P* < 0.05) increased in the GM-applied soils (Table 2), indicating the agricultural management practices play an essential role in regulating the soil metabolite profile. Most of the differentially expressed metabolites are involved in the carbohydrate-, lipid-, and amino acids-related metabolisms (Table 3), all of which are associated with the growth regulation of both microbiota and plants [37]. Considering amino acids-, carbohydrate-, and lipid-related metabolisms are all carbon- or nitrogen-related pathways, this result also suggest that GM treatments clearly improves soil carbon and nitrogen metabolism. Raffinose, melibiose, stachyose, and sucrose are all oligosaccharides involved in galactose metabolism. The oligosaccharides are energetic and structural substances that can serve as carbon sources for plant growth and development [38–40]. Salt stress down-regulated the soil sucrose metabolism and reduced the production of sugars, which adversely affected plant growth [41]. Previous studies have shown that root-secreted malic acid can recruit beneficial bacteria in soil, affect enzymatic activities, and increase phosphorus availability in soil, which benefits plant growth [42, 43]. Moreover, soil application of betaine decreased chromium accumulation and indices of oxidative stress by increasing antioxidant enzymes and metabolites activities in all varieties of sorghum [44]. Rhizosphere soil metabolites analysis also proved that the signal molecule of soil LPC regulated the phosphorus fixation and utilization [45]. The sugarcane/peanut intercropping system secreted more adenosine and adenine in rhizosphere soil, which increased the soil nutrients and promoted plant growth [43]. Compared with continuous peanut cropping, the addition of GMs secreted more beneficial metabolites (such as raffinose, melibiose, stachyose, betaine, sucrose, adenine, LPC, and malic acid) in the present study, which may help peanut growth, improve stress resistance, and address the challenges associated with continuous peanut cropping.

Soil metabolites reflect important metabolic pathways of soil microbial communities, and their concentration are closely associated with the abundances of rhizosphere bacteria [22]. A strong interrelationships of soil metabolites with soil bacterial taxa was identified in this study (Fig. 4). Importantly, the nitrogen cycle-related Actinobacteria and Acidobacteria is highly correlated with lipid-related metabolite 5-Dehydroavenasterol (Fig. 4), providing new evidences to the hypothesis that the soil metabolites may be secreted by microbes [22]. In addition, soil metabolites exuded by plant roots can also reshape the microbial community through root secretions [46–48]. Raffinose has been shown to positively affect the root colonization of specific plant growth-promoting rhizobacteria (PGPRs) [49]. Sucrose repressed the growth of causative fungal pathogen *Fusarium* spp. [50]. Selenium can also shift the rhizosphere bacterial species and population [51]. Therefore, theses up-regulated soil metabolites in GM-applied soils (Table 2) may change the abundance and diversity of bacterial communities. The close interplay of soil bacteria and metabolites displayed in this study was similar to the previous study in Testosterone and PAH-polluted soils [22, 52].

As expected, GMs serve as nutrient sources indeed improved the soil productivity (Fig. 5d-f). In addition, the elevation of carbon and nitrogen cycle-related bacteria containing Actinobacteria, Acidobacteria, and *Sphingomonas* (Fig. 2), and carbon- or nitrogen-related soil metabolites including raffinose, melibiose, stachyose, 3-dehydrosphinganine, betaine, trimethylselenonium, sucrose, 2-methoxyestradiol-17beta 3-sulfate, adenine, LPC, and malic acid (Table 2) in GM-applied soils may be also contributed to the increase of soil available carbon and nitrogen contents, which needs to be further explored. The combination of soil bacteria, metabolomics analysis, soil enzyme activities, and chemical properties can provide comprehensive insight into the advantages of the application of GMs in peanut continuous cropping systems. However, the risks and disadvantages of GMs may also appear, including the additional costs and labor requirements for establishment and regular maintenance, the increased risk of spring frost and crop damage from increased rodent populations, as well as the production of more soil greenhouse gas (such as CH_4_, CO_2_, and N_2_O emissions) [53, 54]. How to give full play to advantages and avoid disadvantages need to be further study. Planting winter wheat as GMs in an optimized way in winter follow period may be a suitable soil management practice to overcome continuous cropping obstacle under continuous spring peanut production systems.

## Conclusions

GM is commonly sown during fallow periods and then chopped and incorporated with soil. In this study, GM treatments improved soil quality and peanut pod yield by shaping the rhizosphere bacterial community and regulating soil metabolites under continuous spring peanut production systems. Comparably, winter wheat was superior to oilseed rape in overcoming continuous cropping obstacle and improving peanut yields, which may be associated with the supplementation of more nitrogen source and carbon and nitrogen cycle-related bacteria. We propose that GM application with winter wheat may be a suitable soil management practice to overcome continuous cropping obstacle under continuous spring peanut production systems.

## Materials and methods

### Experimental design and GMs planting

Field experiments were performed over two winter fallows and two peanut growing seasons (October 2018 to October 2020) at the experimental station of Laixi, Qingdao, China (120.53°E, 36.86°N). The experimental site has a typical temperate monsoon climate, with the average monthly temperature and precipitation shown in Fig. S3. The peanut cultivar cv. Huayu25 provided by Prof. Jing Chen (Shandong Institution of Peanut, http://www.sdshss.com/), was used as the experimental materials in this study. Seeds were sown in the experimental plots on May 10, 2019 and May 6, 2020, and were harvested on October 9, 2019 and October 8, 2020, respectively. Two seeds were planted in each hole, with 25 cm row-spacing and 16.7 cm seed-spacing, and the peanut was hole-sown at a density of 150,000 hills hm^−2^. Triple compound fertilizer 15–15–15 (N-P_2_O_5_-K_2_O) at 600 kg hm^−2^ was incorporated into the soils before peanut planting.

Randomized block design with three replications was used. In northern China, the peanut growing period extends from May to October, and the winter fallow is usually bare fallow [7]. This experiment included the following four treatments: (i) bulk soil without peanut planting (CK); (ii) continuous peanut cropping (CC); (iii) planting winter wheat as GMs in winter fallow period after peanut harvesting (WW); (iv) planting oilseed rape as GMs in winter fallow period after peanut harvesting (OR). The peanut continuous cropping named as CC in this study refers to a system in which peanut is cultivated in the same soil from May to October year after year, and the winter fallow was conventional bare fallow during which no tillage was performed. The experimental groups of GM treatments including winter wheat and/or oilseed rape were applied in winter fallow period after harvesting the peanuts from the previous growing season. Winter wheat cultivar cv. Yannong1212 provided by Prof. Fengbo Wang (Crop Research Institute, Shandong Academy of Agricultural Sciences, http://www.saas.ac.cn/) and oilseed rape cultivar cv. Jiyou1 provided by Prof. Lin Li (Hunan Agricultural University, https://www.hunau.edu.cn/), were used as the experimental materials in this study. Winter wheat was sown on October 23, 2018 and October 29, 2019 at a seeding rate of 240 kg hm^−2^ until early April of the next year, respectively. Oilseed rape was sown on October 25, 2018 and October 30, 2019 at a seeding rate of 60 kg hm^−2^ until early April of the next year, respectively. And then winter wheat and oilseed rape were chopped into 2–4 cm pieces and incorporated manually to a depth of 30 cm. No fertilizer was applied during the wheat- and oilseed rape-growing period.

### Soil sampling and DNA extraction

Rhizosphere soils at peanut maturate period consisted of soils around the roots and root surface soils were sampled as described previously [1]. Rhizosphere soils around the roots: the sampled peanuts were shaken vigorously to remove loose soil and 1–10 mm of soils around the roots were brushed off with a sterile brush [36, 55]. Root surface soils: peanut roots were placed in a centrifuge tube containing 40 mL PBS buffer (pH 7.0, per liter 6.33 g of NaH_2_PO_4_·H_2_O, 16.5 g of Na_2_HPO_4_·7H_2_O, 200 mL Silwet L-77) to extract the root surface soil. The sampled rhizosphere soils were rapidly frozen in liquid nitrogen and stored at −80℃. All the experiments were performed with six replicates. PowerSoilfi DNA Isolation Kit (MoBio Laboratories, Carlsbad, CA, USA) was used to extract soil genomic DNA.

### 16S rRNA gene sequencing

The quality and concentration of the extracted soil genomic DNA was checked by agarose gel electrophoresis and ultraviolet spectrophotometry [56]. 16S rRNA gene sequencing was performed using the specific primers 338F (forward primer, 5′-ACTCCTACGGGAGGCAGCA-3′) and 806R (reverse primer, 5′-GGACTACHVGGGTWTCTAAT-3′) by Shanghai Majorbio (Shanghai, China). The sequencing libraries were produced using TruSeq® DNA PCR-Free Sample Preparation Kit (Illumina, San Diego, CA, USA) and then sequenced on an Illumina HiSeq2500 platform, resulting in 250 bp/300 bp paired-end reads. Paired-end reads were merged using FLASH (version 1.2.7). Operational taxonomic units (OTUs) were clustered at 97% similarity by USEARCH [57].

### Soil bacterial community structure analysis

Alpha diversity analysis containing rarefaction curves which can evaluate the species richness and sequence depth, and rank abundance curves which can reflect the species abundance and evenness (http://en.wikipedia.org/wiki/Rank_abundance_curve) were performed in Majorbio online analysis platform [1]. Beta diversity analysis, including PCoA analysis and ANOSIM analysis performed with 999 displacement tests to detect the statistically significant of the difference were conducted according to the previous study [36]. The phylogenetic tree was constructed using the FastTree tool according to evolutionary relationships [58].

### Soil chemical analysis and soil enzyme activity assays

Three replicates of rhizosphere soil samples of different stages were collected and used for analyses of soil chemical indexes including total carbon contents, available nitrogen contents, and available phosphorous contents [7]. The soil organic carbon was measured by humid oxidation with potassium dichromate (K_2_Cr_2_O_7_), available nitrogen was extracted by using the alkaline hydrolysis diffusion method, and available phosphorus was determined by sodium bicarbonate (NaHCO_3_) spectrophotometry [7]. Soil enzyme activities including invertase, urease, and neutral phosphatase were measured according to the previous study [3]. Soil invertase activity was determined using the 3, 5-dinitrosalicylic acid colorimetric spectrophotometry (absorbance at 508 nm); whereas urease activity was determined using sodium phenolate and sodium hypochlorite spectrophotometry (absorbance at 578 nm); soil neutral phosphatase activity was examined by using Solarbio Soil Neutral Phosphatase (S-NP) Activity Assay Kit (BC0465) [3].

### GC–MS based soil nontargeted metabolomics

Different soil samples were sent to Majorbio for nontargeted metabolomics analysis. The detailed protocol of nontargeted metabolomics was described in a previous study [22]. About 1,000 mg soil were extracted with 1.3 mL of methanol/water (4:1, v/v) solution in a vortex mixer at 4℃ for 30 min. The extract was centrifuged at 13,000 g for 15 min, and the supernatant was re-extracted and re-centrifuged according to the above steps. After three times of extraction, the supernatant was mixed and concentrated under nitrogen gas before LC–MS/MS analysis. Chromatographic separation of metabolites was performed on an ExionLCTMAD system (AB Sciex, USA) having an ACQUITY UPLC BEH C18 column (100 × 2.1 mm; 1.7 mm; Waters, Milford, USA). The UPLC system was coupled to a quadrupole time-of-flight mass spectrometer (Triple TOFTM5600þ, AB Sciex, USA) equipped with an electrospray ionization source that operates in positive and negative mode to perform LC–MS/MS analysis. The equal volumes of all samples are mixed as pooled QC sample for conditioning and quality controlling, which was prepared and tested in the same manner as analytic samples. The data were analyzed using Majorbio online analysis platform following online instructions after normalized with Pareto scaling and log-transformed. KEGG analysis were performed to identify the altered pathway involved in the GMs application. All the pathways mapped by the differential metabolites of soils were compiled, in terms of the KEGG orthology terms (www.kegg.jp/kegg/kegg1.html) in the KEGG pathway database [59–61].

### Measurement of peanut yields and its components

In maturation period, the peanuts were collected in a 6.67 m^2^ (3.335 × 2 m) area (from which no plants were sampled) for yield analysis. Fifteen representative plants were sampled from each treatment to analyze the plant morphology. All samples were heated at 105℃ for 30 min, then dried to a constant weight at 80℃, and counted separately as the previous study [36]. All pods harvested from peanut plants were air-dried and weighed to achieve the 100-pod weight. Moreover, the shells were peeled to obtain 100-pod weight.

### Statistical analysis

All experiments were performed with three times. Error bars in each graph of soil chemical analysis and soil enzyme assays indicate the mean values ± SEM. Statistically significant differences between soil groups were estimated using Student’s *t*-test (*P < 0.05; **P < 0.01; ***P < 0.001) and one-way ANOVA (*P* < 0.05; LSD and Duncan test) with Statistical Product and Service Solutions Statistics software (SPSS 23; IBM).

## Supplementary Information

**Additional file 1:** **Fig S1.** Bacterial community structure at the class, order, and family levels in the rhizosphere and bulk soils. (a) Percent of taxa at the order level in the rhizosphere and bulk soils. The relative abundance of each taxon was calculated by averaging the abundances of three duplicates in each soil group. (b) Percent of taxa at the class level in the rhizosphere and bulk soils. (c) Percent of taxa at the family level in the rhizosphere and bulk soils.

**Additional file 2: Fig S2.** Pathway analysis of the identified differential metabolites. (a) Pathway impact resulting from the differential metabolites using MetaboAnalyst 3.0 between WW and CC. Small p-value and big pathway impact factor indicate that the pathway is greatly influenced. (b) Pathway impact resulting from the differential metabolites using MetaboAnalyst 3.0 between OR and CC. Small p-value and big pathway impact factor indicate that the pathway is greatly influenced.

**Additional file 3:** **Fig S3.** The average monthly temperature and precipitation in the 2018, 2019, and 2020 growing seasons.

**Additional file 4:** **Table S1.** The number of identified metabolites and classified in the Kyoto Encyclopedia of Genes and Genomes (KEGG).

**Additional file 5:** **Table S2.** The identified metabolites in the KEGG pathway.

## Data Availability

All raw sequences for 16S rRNA gene sequencing are available in the NCBI Sequence Read Archive with accession number BioProject PRJNA849950 (https://ncbi.nlm.nih.gov/bioproject/849950) and all raw sequences for nontargeted metabolomics are available in the CNGB Sequence Archive (CNSA) of China National GeneBank DataBase (CNGBdb, https://db.cngb.org) with accession number CNP0003663 (https://db.cngb.org/search/project/CNP0003663/).

## Abbreviations

GM: Green manure
PGPRs: Plant growth-promoting rhizobacteria
CK: Bulk soil without peanut planting
CC: Continuous peanut cropping
WW: Planting winter wheat as GMs in winter fallow period after peanut harvesting
OR: Planting oilseed rape as GMs in winter fallow period after peanut harvesting
PCoA: Principal co-ordinates analysis
ANOSIM: Analysis of similarities
QC: Quality control
LPC: Lysophosphatidylcholine
KEGG: Kyoto encyclopedia of genes and genomes
VIP: Variable importance in projection
PLS-DA: Partial least-squares discrimination analysis
