# Supplementary material for: Green manure increases peanut production by shaping the rhizosphere bacterial community and regulating soil metabolites under continuous peanut production systems
Source: BMC Plant Biol. 2023 Feb 1;23:69. doi: 10.1186/s12870-023-04079-0 (PMC9890850; doi:10.1186/s12870-023-04079-0)
Supplement: Supplementary file 1 — Additional file 1: Fig S1. Bacterial community structure at the class, order, and family levels in the rhizosphere and bulk soils. (a) Percent of taxa at the order level in the rhizosphere and bulk soils. The relative abundance of each taxon was calculated by averaging the abundances of three duplicates in each soil group. (b) Percent of taxa at the class level in the rhizosphere and bulk soils. (c) Percent of taxa at the family level in the rhizosphere and bulk soils. [file 12870_2023_4079_MOESM1_ESM.pdf]

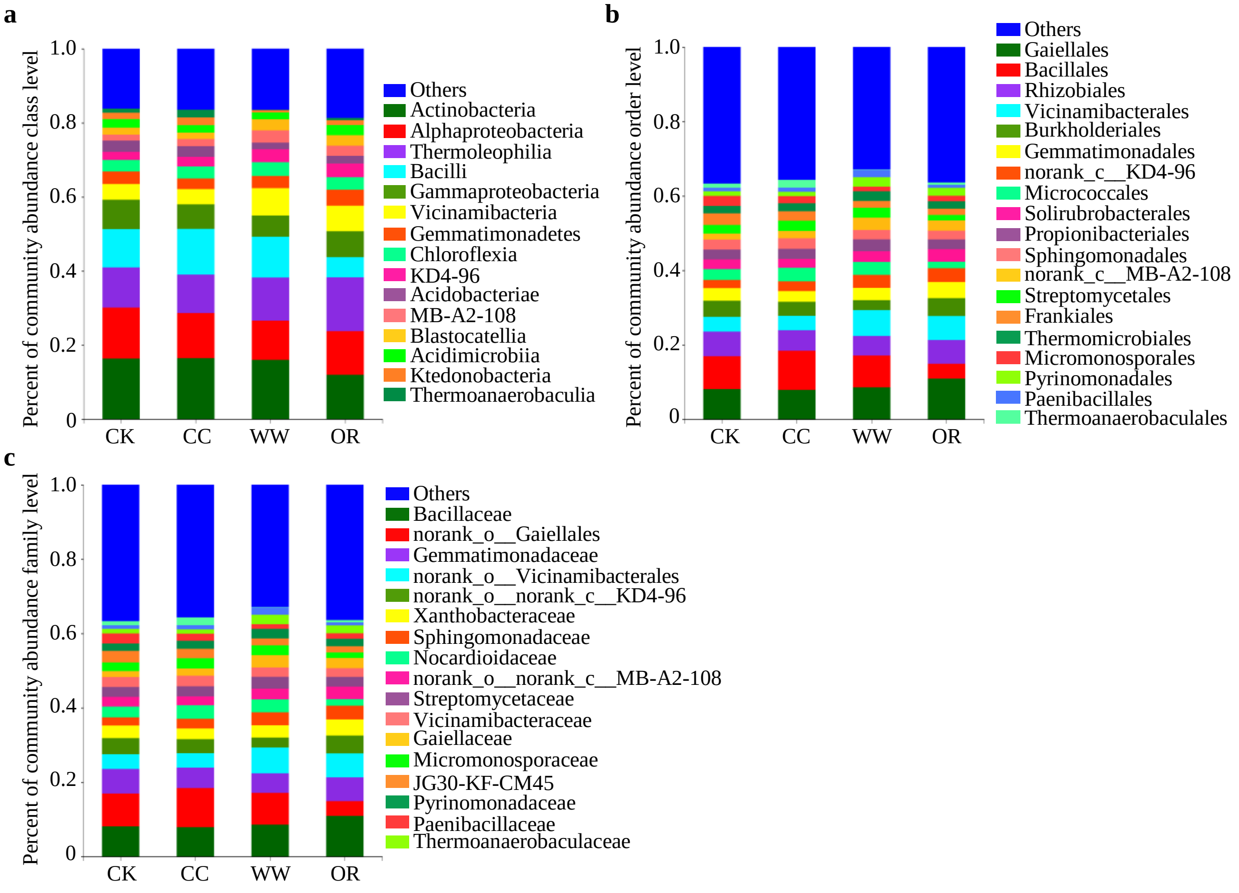

Additional file 1 Fig. S1 Bacterial community structure at the class, order, and family levels in the rhizosphere and bulk soils. (a) Percent of taxa at the order level in the rhizosphere and bulk soils. The relative abundance of each taxon was calculated by averaging the abundances of three duplicates in each soil group. **(b)** Percent of taxa at the class level in the rhizosphere and bulk soils. **(c)** Percent of taxa at the family level in the rhizosphere and bulk soils.
