# Supplementary material for: Green manure increases peanut production by shaping the rhizosphere bacterial community and regulating soil metabolites under continuous peanut production systems
Source: BMC Plant Biol. 2023 Feb 1;23:69. doi: 10.1186/s12870-023-04079-0 (PMC9890850; doi:10.1186/s12870-023-04079-0)
Supplement: Supplementary file 2 — Additional file 2: Fig S2. Pathway analysis of the identified differential metabolites. (a) Pathway impact resulting from the differential metabolites using MetaboAnalyst 3.0 between WW and CC. Small p-value and big pathway impact factor indicate that the pathway is greatly influenced. (b) Pathway impact resulting from the differential metabolites using MetaboAnalyst 3.0 between OR and CC. Small p-value and big pathway impact factor indicate that the pathway is greatly influenced. [file 12870_2023_4079_MOESM2_ESM.pdf]

a

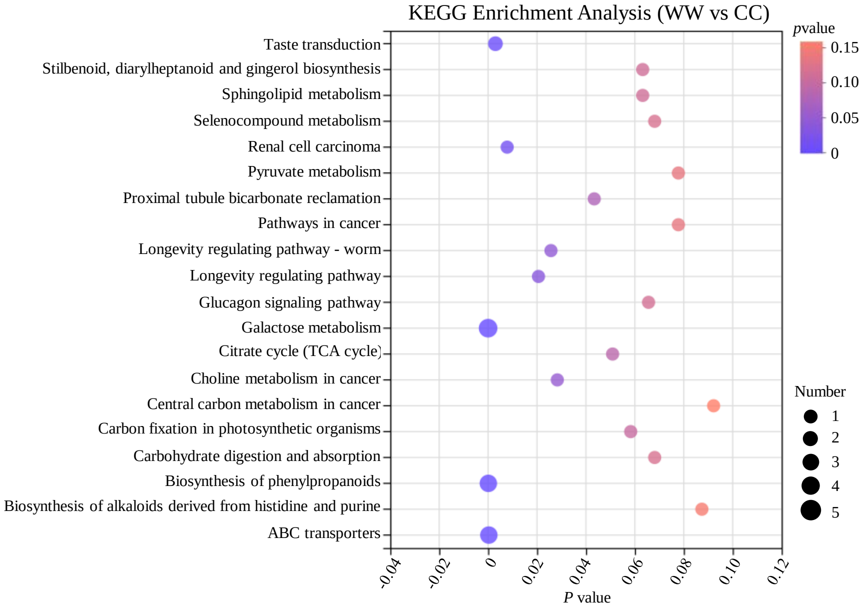

b

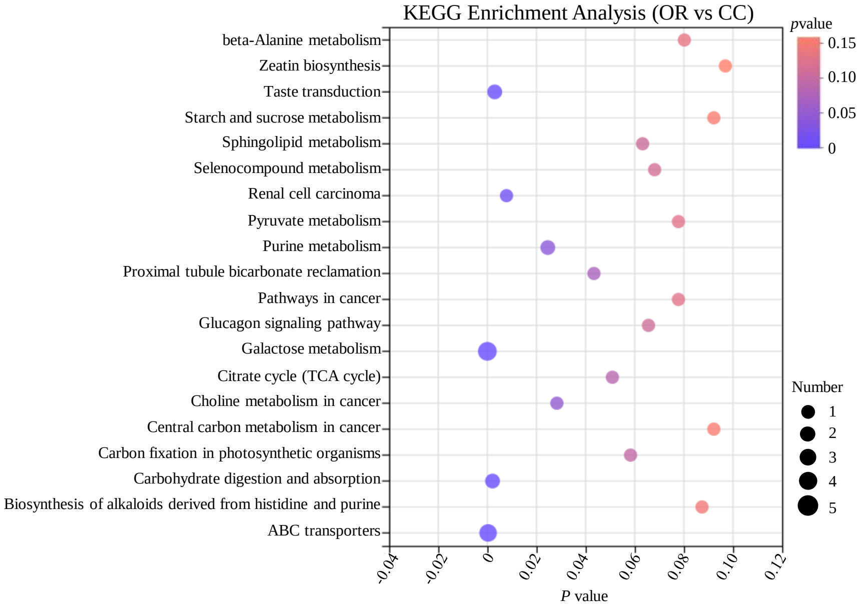

Additional file 2 Fig. S2 Pathway analysis of the identified differential metabolites. (a) Pathway impact resulting from the differential metabolites using MetaboAnalyst 3.0 between WW and CC. Small *p*-value and big pathway impact factor indicate that the pathway is greatly influenced. **(b)** Pathway impact resulting from the differential metabolites using MetaboAnalyst 3.0 between OR and CC. Small *p*-value and big pathway impact factor indicate that the pathway is greatly influenced.
