# Supplementary material for: Green manure increases peanut production by shaping the rhizosphere bacterial community and regulating soil metabolites under continuous peanut production systems
Source: BMC Plant Biol. 2023 Feb 1;23:69. doi: 10.1186/s12870-023-04079-0 (PMC9890850; doi:10.1186/s12870-023-04079-0)
Supplement: Supplementary file 3 — Additional file 3: Fig S3. The average monthly temperature and precipitation in the 2018, 2019, and 2020 growing seasons. [file 12870_2023_4079_MOESM3_ESM.pdf]

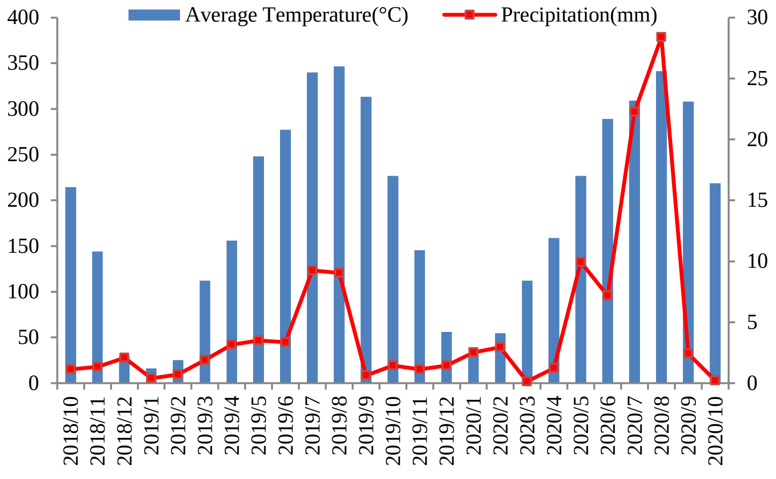

Additional file 3 Fig. S3 The average monthly temperature and precipitation in the 2018, 2019, and 2020 growing seasons.
