# Supplementary material for: Green manure increases peanut production by shaping the rhizosphere bacterial community and regulating soil metabolites under continuous peanut production systems
Source: BMC Plant Biol. 2023 Feb 1;23:69. doi: 10.1186/s12870-023-04079-0 (PMC9890850; doi:10.1186/s12870-023-04079-0)
Supplement: Supplementary file 4 — Additional file 4: Table S1. The number of identified metabolites and classified in the Kyoto Encyclopedia of Genes and Genomes (KEGG). [file 12870_2023_4079_MOESM4_ESM.pdf]

Additional file 4 Table S1 The number of identified metabolites and classified in the Kyoto Encyclopedia of Genes and Genomes (KEGG)

Ion mode	All peaks	Identified metabolites	Metabolites in KEGG
pos	4001	260	68
neg	3368	198	40
