# Supplementary material for: Green manure increases peanut production by shaping the rhizosphere bacterial community and regulating soil metabolites under continuous peanut production systems
Source: BMC Plant Biol. 2023 Feb 1;23:69. doi: 10.1186/s12870-023-04079-0 (PMC9890850; doi:10.1186/s12870-023-04079-0)
Supplement: Supplementary file 5 — Additional file 5: Table S2. The identified metabolites in the KEGG pathway. [file 12870_2023_4079_MOESM5_ESM.pdf]

Additional file 5 Table S2 The identified metabolites in the KEGG pathway

Metabolite	KEGG Pathway ID	KEGG Pathway Description
Vestitone	map00943;map01110	Isoflavonoid biosynthesis; Biosynthesis of secondary metabolites
Limonene-1,2-epoxide	map00903	Limonene and pinene degradation
Geranylgeraniol	map01110;map00904	Biosynthesis of secondary metabolites; Diterpenoid biosynthesis
Nerolidol	map01100;map01062;map00909	Metabolic pathways; Biosynthesis of terpenoids and steroids; Sesquiterpenoid and triterpenoid biosynthesis
(1R,4R)-Dihydrocarvone	map01220;map00903	Degradation of aromatic compounds; Limonene and pinene degradation
OXCARBAZEPINE	map00982	Drug metabolism - cytochrome P450
Daidzein	map00943;map01110;map01061	Isoflavonoid biosynthesis; Biosynthesis of secondary metabolites; Biosynthesis of phenylpropanoids
2-Methoxyestradiol-17beta 3-sulfate	map00140	Steroid hormone biosynthesis
Sphinganine	map00600;map01100;map04071	Sphingolipid metabolism; Metabolic pathways; Sphingolipid signaling pathway
(-)-Carvone	map01110;map00903;map00902	Biosynthesis of secondary metabolites; Limonene and pinene degradation; Monoterpenoid biosynthesis
Leukotriene A4	map00590;map01100;map07034;map04726	Arachidonic acid metabolism; Metabolic pathways; Eicosanoids; Serotonergic synapse
P-Tolualdehyde	map01100;map01220;map00622;map01120	Metabolic pathways; Degradation of aromatic compounds; Xylene degradation; Microbial metabolism in diverse environments
Nobiletin	map00941	Flavonoid biosynthesis
Betaine	map01100;map02010;map00260	Metabolic pathways; ABC transporters; Glycine, serine and threonine metabolism
Sucrose	map01100;map01110;map04973;map02010;map00052;map00500;map02060;map04742	Metabolic pathways; Biosynthesis of secondary metabolites; Carbohydrate digestion and absorption; ABC transporters; Galactose metabolism; Starch and sucrose metabolism; Phosphotransferase system (PTS); Taste transduction
Hypoxanthine	map01100;map00230	Metabolic pathways; Purine metabolism
Coumarin	map01110;map00940	Biosynthesis of secondary metabolites; Phenylpropanoid biosynthesis
M-Methylbenzoate	map01100;map01220;map00622;map01120	Metabolic pathways; Degradation of aromatic compounds; Xylene degradation; Microbial metabolism in diverse environments
16-Hydroxy hexadecanoic acid	map01100;map00073	Metabolic pathways; Cutin, suberine and wax biosynthesis
13(S)-HOTrE	map00592	alpha-Linolenic acid metabolism
2-Indanone	map00624;map01120	Polycyclic aromatic hydrocarbon degradation; Microbial metabolism in diverse environments
3-Aminopropanal	map01100;map00410	Metabolic pathways; beta-Alanine metabolism
P-Salicylic acid	map01100;map01220;map01110;map01120;map00627;map00130;map00623;map00363;map00362;map00790;map07110;map01061	Metabolic pathways; Degradation of aromatic compounds; Biosynthesis of secondary metabolites; Microbial metabolism in diverse environments; Aminobenzoate degradation; Ubiquinone and other terpenoid-quinone biosynthesis; Toluene degradation; Bisphenol degradation; Benzoate degradation; Folate biosynthesis; Benzoic acid family; Biosynthesis of phenylpropanoids
5-Dehydroavenasterol	map01110;map00100	Biosynthesis of secondary metabolites; Steroid biosynthesis
M-Cresol	map01100;map01220;map00633;map01120;map00623;map04974	Metabolic pathways; Degradation of aromatic compounds; Nitrotoluene degradation; Microbial metabolism in diverse environments; Toluene degradation; Protein digestion and absorption
N-Ethyl trans-2-cis-6-nonadienamide	map01100;map01120;map00791	Metabolic pathways; Microbial metabolism in diverse environments; Atrazine degradation
Gamma-Tocotrienol	map01100;map00130;map01110	Metabolic pathways; Ubiquinone and other terpenoid-quinone biosynthesis; Biosynthesis of secondary metabolites
7,8-Dihydroneopterin	map01100;map00790	Metabolic pathways; Folate biosynthesis
1,2,3-Trihydroxybenzene	map00627;map07216;map01120	Aminobenzoate degradation; Catecholamine transferase inhibitors; Microbial metabolism in diverse environments
Styrene	map01100;map01220;map01120;map00642;map00643	Metabolic pathways; Degradation of aromatic compounds; Microbial metabolism in diverse environments; Ethylbenzene degradation; Styrene degradation
(-)-Medicarpin	map00943;map01110;map01061	Isoflavonoid biosynthesis; Biosynthesis of secondary metabolites; Biosynthesis of phenylpropanoids
Anthracene-9,10-dihydrodiol	map00624;map01120	Polycyclic aromatic hydrocarbon degradation; Microbial metabolism in diverse environments
Resveratrol	map04212;map04211;map01110;map01061;map00945	Longevity regulating pathway - worm; Longevity regulating pathway; Biosynthesis of secondary metabolites; Biosynthesis of phenylpropanoids; Stilbenoid, diarylheptanoid and gingerol biosynthesis
3-Isochromanone	map00624;map01120	Polycyclic aromatic hydrocarbon degradation; Microbial metabolism in diverse environments
